# Chiral Rare Earth Nanomaterials: Synthesis, Optical Properties, and Potential Applications

**DOI:** 10.3390/nano15171321

**Published:** 2025-08-28

**Authors:** Lei Zhao, Pan Liang, Hua Zhao, Rongrong Hu, Yangyang Xu, Fangfang Chen, Xianghu Wang, Yunhua Yao

**Affiliations:** 1 School of Arts and Sciences, Shanghai Dianji University, Shanghai 200240, China; 2 College of Sciences, Shanghai Institute of Technology, Shanghai 201418, China; 3 State Key Laboratory of Precision Spectroscopy, East China Normal University, Shanghai 200241, China

**Keywords:** chirality, rare earth nanomaterials, circularly polarized luminescence

## Abstract

Chiral rare earth nanomaterials, which impart optical activity through chiral structures or ligands, possess promising application potential in optoelectronic displays, biosensing, information encryption, and catalysis due to unique properties like circularly polarized luminescence, exceptional photostability, and tunable optics. This review systematically summarizes recent advancements, focusing on synthetic strategies, distinctive optical properties, and experimental demonstrations of applications across various fields. Finally, prospects and challenges for future development are discussed. These studies advance the understanding of circularly polarized luminescence and enable the flexible design and fabrication of chiral rare earth nanomaterials with engineered functionalities, addressing practical challenges in optoelectronic displays and biomedicine.

## 1. Introduction

Chirality is a property of an object or molecule that cannot be superimposed onto its mirror image [1,2]. Chirality widely exists in nature and can be observed across all scales, from smallest subatomic particles to vast galactic structures. The study of this property is of great significance in fields such as pharmaceutical, bio-pharmaceutical, food, and cosmetic industries [3]. In materials science, chirality influences not only the optical activity of chiral nanomaterials, but also their physical and chemical properties, including electronic spin and catalytic selectivity [4,5,6]. At the nanoscale, the effects of chirality are often significantly amplified, leading to novel phenomena that are absent in bulk materials. For instance, self-assembly is a unifying characteristic of nanoscale particles, enabling the translation of chirality from molecular and nanometer scales to submicron and micrometer scales. This multiscale range allows for the observation of various electronic, chemical, optical, and biological effects, which is essential to engineering sensors as well as photonic, optoelectronic, and information technologies [7]. With the scientific advancement in the fields of nanoscience and chirality, a wide variety of chiral nanomaterials are synthesized and self-assembled, such as chiral quantum dots [8,9,10,11], chiral perovskites [12,13,14,15], and chiral nanocomposites [16]. In recent years, chiral nanomaterials have garnered significant attention in fields such as biology, chemistry, life sciences, chiral nano photonics, and metamaterials [17,18,19,20]. This growing interest can be largely attributed to the introduction of chiral degrees of freedom, which enhance the properties of materials at the nanoscale. The optical properties of chiral nanomaterials can be precisely controlled by manipulating their sizes and shapes.

Rare earth elements consist of 15 lanthanide elements, and the elements Scandium (Sc) and Yttrium (Y). Lanthanide elements have unique electron configurations of [Xe] 4f^n−1^5d^0−1^6s^2^ (n = 1–15), and the trivalent lanthanide ions (Ln^3+^) are the most common and stable despite having several chemical valences [21]. The unpaired 4f electrons in rare earth elements are shielded by outer 5s and 5p orbitals, making them relatively insensitive to crystal field effects. Compared to that, transition metals like Cu and Co have partially filled d orbitals [22], which have broader and less defined electronic transitions, rare earth elements have sharp, well-defined electronic transitions. Due to the unique 4f energy levels, rare earth nanomaterials have the advantages of excellent photostability, a large anti-Stokes shift, long luminescence lifetime, and sharp-band emission [23]. Owing to their unique optical properties, Ln^3+^ doped luminescent nanocrystals are promising for applications ranging from biosensor, lasing, photovoltaic devices, information security, and electrocatalysis [23,24,25].

Chiral rare earth nanomaterials not only exhibit the inherent advantages of rare earth elements but also introduce optical activity through their chiral structures or chiral ligands. This combination imparts unique application potential in optical sensors, biological labeling, and next-generation display technology [26,27,28,29]. Chiral nano-sized rare earth materials demonstrate significant circular dichroism (CD) and circularly polarized luminescence (CPL) properties. CD and CPL spectroscopies are the typical technical means for characterizing the optical activity of chiral materials. CD spectroscopy can precisely analyze the ground-state optical properties of materials by detecting the absorption difference in left- and right- circularly polarized light, providing key evidence for processes such as chiral ligand modification, nanoparticle self-assembly, or lattice symmetry breaking. CPL spectroscopy directly reflects the chiral information of the excited state in chiral luminophores or phosphors by recording the intensity difference in left- and right- circularly polarized light during the luminescence process, and it is particularly suitable for studying the f-f transition characteristics of rare earth ions (such as Eu^3+^, Tb^3+^, etc.). The generation mechanism of CPL involves the emission of a photon from a chiral excited state, which gains polarization due to a nonzero dot product between the electric dipole transition moment and the imaginary magnetic dipole transition moment, characterizing the electronic transition responsible for luminescence [30].

The research on chiral rare earth materials is expected to expand the application range of CPL from the visible light region to the ultraviolet (UV) and near-infrared regions [31], possessing high stability [32,33]. For example, cerium (Ce)-based rare earth metal halides, such as (R/S-MBA)CeCl_4_·2CH_3_OH (MBA = α-methylbenzylammounium), exhibit pronounced CD in the ultraviolet region (340–400 nm) and display distinct CPL characteristics in the visible light region (300–700 nm), expanding the application potential of chiral optoelectronics (such as UV-CPL light sources) [31]. Furthermore, by modifying the luminescent ions incorporated into the lattice, color-tunable CPL can be achieved, and this type of CPL exhibits excellent thermal stability and can maintain luminescence at temperatures exceeding 300 °C [32]. Chiral rare earth fluoride nanoparticles induced by helical silica can still maintain their CPL activity even after calcination at 400 °C [33]. This resistance to high temperatures enables encryption and display applications in high-temperature environments. By doping Er^3+^/Yb^3+^ ions into bismuth oxychloride-based inorganic nanostructured phosphor, modified with chiral sugar alcohols, exhibiting enhanced upconversion luminescence compared to non-chiral phosphors [34], and thus expanding its application in biological imaging and photodynamic therapy. These advancements establish a solid foundation for practical applications of chiral rare earth nanomaterials.

**Figure 1:**
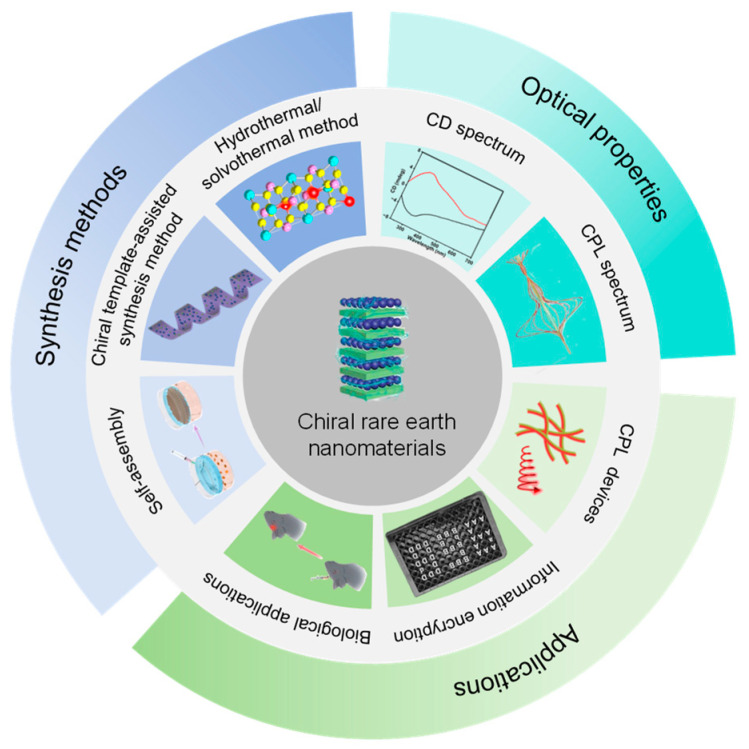
Synthesis methods, optical properties, and application of chiral rare earth nanomaterials.

Recent reviews have summarized the advances in chiral rare earth nanomaterials [29,35,36], but this work sets itself apart by proposing a unified framework for understanding the origins of chirality and synthesis methods and incorporating groundbreaking applications such as stimulus-responsive circularly polarized luminescence and high-security encryption that have been reported in 2024–2025. This article provides a comprehensive overview of recent advancements in chiral rare earth nanomaterials, focusing on three critical aspects: synthesis strategies, optical properties, and potential applications (Figure 1). We begin by exploring the origins of chirality in chiral rare earth nanomaterials and categorizing synthesis strategies into three primary methods: hydrothermal/solvothermal methods, chiral template-assisted synthesis, and self-assembly. Next, we present the optical properties, including CD and CPL. Given the unique optical characteristics of chiral rare earth nanomaterials, we then discuss their potential applications in circularly polarized light-emitting devices, optical anti-counterfeiting and information encryption, as well as drug delivery, therapy, and bioimaging. Finally, we highlight current challenges and future perspectives in this rapidly evolving field.

## 2. The Origin of Chirality

**Figure 2:**
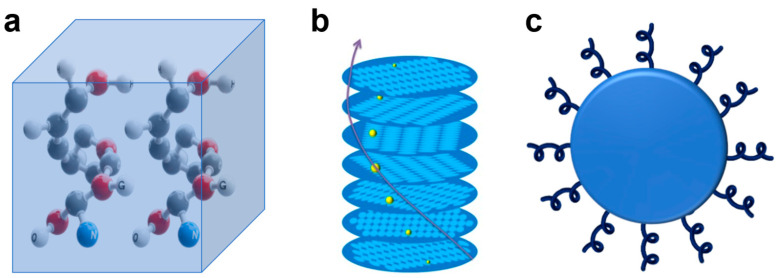
Schematic diagram of chirality origin of chiral rare earth nanomaterials. (**a**) Intrinsically chiral crystal. (**b**) Co-assembly between nanomaterials and a chiral template. (**c**) Chiral transfer between inorganic nanomaterials and chiral ligands.

The crystalline structure, shape, and interaction of chiral ligands are the factors that result in chirality in inorganic nanomaterials [37]. For semiconductor nanocrystals, the origins of chirality are well known and can be classified into three types: (1) semiconductor nanocrystal core containing dislocations and defects, (2) incorporation and arrangement of nanocrystals into chiral superstructures, and (3) interactions between chiral ligands and the nanocrystal [38]. In strict analogy with the classification in semiconductor nanocrystals, chirality in chiral rare earth nanomaterials originate from (1) intrinsic chirality arising from chiral crystal, (2) chiral assemblies of achiral rare earth nanomaterials, and (3) ligand-induced chirality into nanomaterials. According to their chiral sources, there are intrinsic chiral rare earth nanomaterials, chiral co-assembled rare earth nanomaterials, and ligand-induced chiral rare earth nanomaterials, respectively, as shown in Figure 2.

In intrinsic chiral rare earth nanomaterials, chirality is determined by the inherent structure at the atomic scale, such as the inherent chiral space group of the crystal structure. Chiral objects or molecules exist in two non-overlapping forms in space, known as enantiomers [39]. The enantiomer with molecular chirality is used to guide the symmetry breaking of the chiral nanocrystals, resulting in a preferential chiral space group [40]. The resultant nanocrystals are categorized as L- or D-form based on the molecular enantiomer, and X-ray diffraction can verify that the sample’s chirality originates from the chiral lattice. LaPO_4_:RE^3+^ (RE = Eu, Tb, Tm) nanowires are grown in a direction controlled by chiral molecular inducers, forming nanoscale structures with atomic-scale chirality [32,41]. These nanomaterials, regardless of their external shapes, have chiral lattice arrangements internally and can interact with polarized light [37]. These intrinsically chiral rare earth nanomaterials are more stable than other assemblies, showing clear CPL signals even at temperatures over 300 °C [32].

Interestingly, nanoscale chirality can also be achieved by co-assembly of achiral nanomaterials with chiral templates such as DNA [42,43,44], liquid crystal [35,45,46,47], chiral polymer [48,49], and organogelators [50,51,52]. In chiral co-assembled rare earth nanomaterials, chiral templates (such as liquid crystals, helical silica, etc.) are utilized as hosts to guide the formation of chiral structures, where rare earth materials are used as guests. For example, HS@CeF_3_:RE (HS = helical silicas) nanocomposites synthesized using helical silica templates [33], and chiral luminescent nanomaterials prepared by liquid crystal templates [35]. These chiral assemblies of achiral rare earth nanomaterials are expected to be useful for studying their interparticle coupling and responsive dynamic properties, which could lead to a wide range of potential applications [35,53,54].

Interaction with chiral ligands or molecules is another strategy to induce chirality in nanomaterials. Ligand exchange between chiral ligands and initial achiral ligands is a commonly used method to induce chirality in chalcogenide metal semiconductors, such as CdS and CdSe quantum dots [10,55]. This process does not destroy the core structure of the quantum dots. In ligand-induced chiral rare earth nanomaterials, chirality can also arise from interactions with chiral ligands such as amino acids or short peptides. Unlike the ligand-exchange method in quantum dots, Dawn E. Barry et al. designed near-infrared lanthanide-emissive Langmuir−Blodgett monolayers using Nd (III) directed self-assembly synthesis of chiral amphiphilic ligands [56]. These ligands impart both amphiphilicity and chirality to the complexes.

## 3. Synthesis Methods

According to the origin of chirality in chiral rare earth nanomaterials, we classify the synthetic methods into three categories: solvothermal method, template-assisted co-assembly, and self-assembly method. The first method synthesizes intrinsic chiral rare earth nanomaterials, while the latter two methods synthesize non-intrinsic chiral rare earth nanomaterials. The intrinsic chiral rare earth nanomaterials usually have higher stability than the non-intrinsic chiral rare earth nanomaterials due to the atomic chirality. In the following, we will elaborate on the synthetic methods of chiral rare earth nanomaterials in detail.

### 3.1. Hydrothermal/Solvothermal Method

Hydrothermal/solvothermal method has garnered significant attention for its innovative use of chiral molecules as inducers [32,34,41,57]. This method leverages the unique properties of chiral molecules to direct the preferential growth of materials along specific chiral axes. Enantiomers play a critical role in the synthesis and development of chiral nanocrystals, as they precisely guide the process of symmetry breaking. This guidance ensures that the formation of these nanocrystals favors a specific, preferred chiral space group. Consequently, the final nanocrystalline structures, which reflect this directed symmetry breaking, are accurately classified and designated as either L-form or D-form, corresponding directly to the identity of the molecular enantiomer used in their fabrication. This systematic approach not only underscores the significance of enantiomer selection but also illustrates the meticulous control exercised over the chirality of the resulting nanomaterials [40,41].

**Figure 3:**
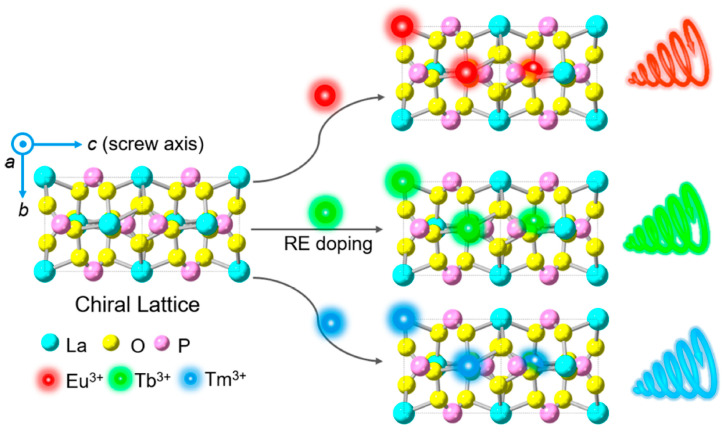
Schematic illustration of the rational design of chiral nanocrystals with atomic-scale chirality for tunable color circularly polarized luminescence emission [32].

Researchers have developed a novel family of intrinsically chiral rare earth nanowires via this facile solution method [32,41], as shown in Figure 3. By incorporating different kinds of rare earth ions (such as europium (Eu^3+^), terbium (Tb^3+^), and thulium (Tm^3+^)), these chiral nanowires emit multicolor CPL (e.g., red, green, or blue). Chiral organic and organic−inorganic hybrid system also possesses color-tunable CPL but suffers from chiral instability employing unstable chiral supramolecules or their dependence on chiral surface ligands [58,59,60]. However, the chiral nanowires synthesized by this method have high thermal stability at high temperatures of up to 300 °C due to the atomic chirality, which could be well coupled with other functional materials. What is more, by injecting this chiral nanowire dispersion into poly (vinyl alcohol) solution and after drying naturally, a substrate-free flexible chiral film is obtained. Thus, this method not only realizes atomic-scale chiral crystals with robust, color-tunable CPL properties but also enables innovative CPL application prospects.

### 3.2. Chiral Template-Assisted Synthesis Method

Compared to intrinsic chiral rare earth nanomaterials, chiral co-assembled rare earth nanomaterials exhibit a broader variety of types. Currently, various systems have been reported that achieve chirality through the co-assembly of non-chiral luminescent guests (rare earth luminescent nanoparticles) and chiral hosts. Typically, the chiral host provides a chiral confined space for the non-chiral luminescent guest. When the luminescent guest assembles within this chiral confined space, chirality can be effectively transferred from the chiral host to the luminescent guest. Due to the diversity in the selection of chiral hosts and luminescent guests, this strategy can achieve tunable optical properties by regulating different luminescent centers and chiral hosts, thereby demonstrating significant versatility. The following sections introduce several common chiral templates, including chiral metal–organic frameworks (MOFs), chiral liquid crystals, chiral organic gels, chiral helical SiO_2_ structures, and chiral layered structures.

#### 3.2.1. Co-Assemble with Chiral MOFs Template

**Figure 4:**
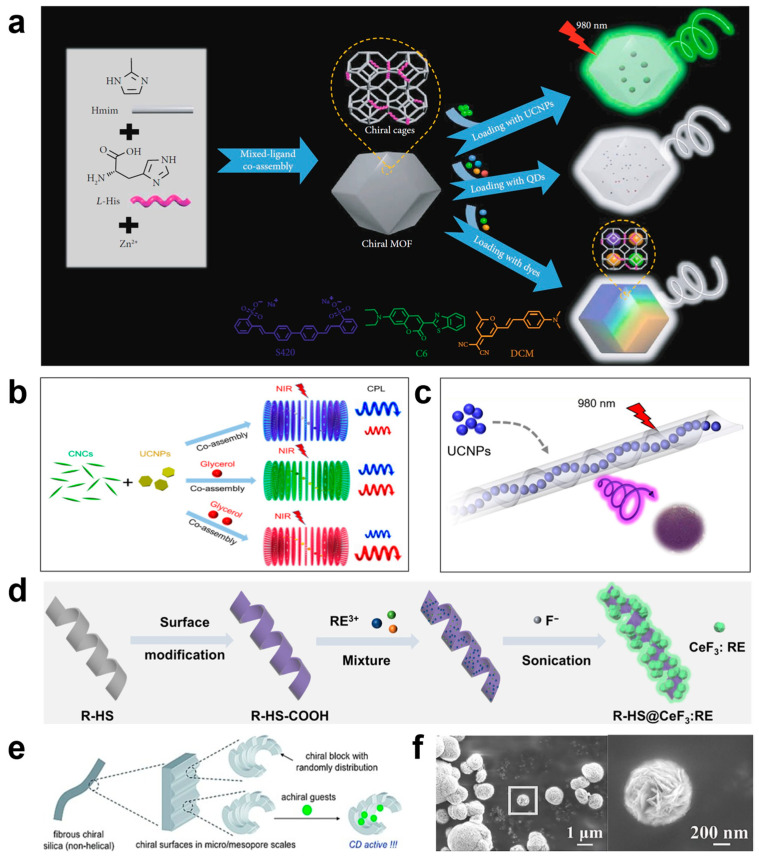
(**a**) Schematic synthesis of chiral MOFs and CPL-active MOFs [65]. (**b**) Illustration showing CNC-based chiral photonic films fabricated via the co-assembly of CNCs, glycerol, and lanthanide-doped UCNPs [74]. (**c**) Schematic representation of upconverted circularly polarized luminescence based on co-gel with UCNPs [26]. (**d**) Schematic illustration for the synthesis of R-HS@CeF_3_: rare earth nanocomposites [33]. (**e**) Illustration showing geometrically organized chiral segments dispersed throughout the silica wall, along with confined chromophores [84]. (**f**) SEM images of D-BYE [34].

MOFs are a subclass of coordination polymers known for their highly ordered crystalline porous structure self-assembled by metal ions and organic ligands [61], which have attracted considerable attention from researchers. Introducing chirality into these MOF materials is expected to confer several advantageous properties, such as asymmetric catalysis and enantioselective recognition or separation [62]. Nanomaterials such as quantum dots and upconversion nanoparticles are easily loaded into the chiral MOFs during the synthetic process. ZIF-8 is a representative zeolitic imidazolate framework (a subclass of MOFs) material with a sodalite topology, formed by zinc ions and 2-methylimidazole (Hmim) [63,64]. The chiral ZIF can be synthesized through the method of mixed-ligand co-assembly [65]. For example, L-histidine (L-His) and Hmim were dissolved in a mixed solvent of methanol and water. A small amount of triethylamine was then added to the solution under stirring. After stirring for 10 min, the resulting mixed-ligand solution was gradually added to a methanol solution of Zn(NO_3_)_2_·6H_2_O. The mixture was stirred at room temperature for 24 h. The colorless product was then washed with large amounts of water and methanol, collected by centrifugation, and dried under vacuum. Various types of launchers, including lanthanide-doped upconversion nanoparticles (UCNPs), can be effectively incorporated into chiral MOFs through a straightforward in situ synthesis approach as shown in Figure 4a [65]. In addition, through the self-assembly of chiral MOFs and perovskite nanocrystals (NCs), Zhang et al. prepared a pair of crystalline enantiomeric adducts, (P)-(+)/(M)-(−)-EuMOF⊃MAPbX_3_ (where MA = CH_3_NH_3_^+^, and X = Cl^−^, Br^−^, I^−^). This co-assembly strategy aims to embed achiral MAPbBr_3_ perovskite NCs within chiral MOFs by inheriting the chirality of the host MOFs through host–guest Eu–Br and Pb–O coordination bonds [58]. These works open a new avenue for the general fabrication of solid-state CPL composite materials [60,66,67].

#### 3.2.2. Chiral Liquid-Crystalline Template-Assisted Synthesis Method

In recent, chiral liquid-crystalline templates have also been used to synthesize chiral nanomaterials [68,69,70,71]. Cholesteric or chiral nematic liquid crystals with helical nanostructures are the most commonly used method to achieve CPL enhancement by using chiral liquid crystal templates. As a chiral template, one of attractive features of chiral nematic liquid crystal is the photonic bandgap effect, which selectively reflects circularly polarized light with the same handedness and transmits light with the opposite direction. It provides a way to control the chiral direction. Cellulose nanocrystals (CNCs), as renewable nanomaterials, can be produced in large quantities through acid hydrolysis of natural fibers [29]. When CNCs are dispersed in water solvents at a certain concentration, they can self-assemble to form left-handed nematic liquid crystals. Self-assembled photonic films made from CNCs can selectively reflect CPL, which is similar to some crustaceans that reflect light through a spiral structure [72,73]. The intriguing properties of CNCs have inspired research into generating CPL by doping fluorescent chromophores into photonic cellulose films, for example, Li et al. obtained CNC-based chiral photonic films fabricated via the co-assembly of CNCs liquid-crystalline template, glycerol, and lanthanide-doped UCNPs as shown in Figure 4b [74]. During the synthesis process, (NaYF_4_:Tm/Yb) UCNPs were first prepared. Polyvinyl alcohol (PVA) was added to an aqueous dispersion of UCNPs and then the mixture was stirred for 1 h at ambient temperature to give the UCNP@PVA dispersion. UCNP@PVA dispersion and different amounts of glycerol were added to the CNCs suspension with vigorous stirring for 5 h, the suspension was cast in a rectangular plastic container and left to dry naturally for 2 to 3 days, thereby obtaining chiral photonic films. Li et al. indicated that with the incorporation of multiple-emissive chromophores into a photonic cellulose film with an adjustable photonic bandgap (PBG), tunable CPL might be achieved simply by adjusting the PBG of chiral host. Thus, by combining a variety of luminescent nanomaterials—such as quantum dots, perovskite nanocrystals, and rare earth nanoparticles—this method provides a convenient and environmentally friendly way to obtain CPL materials.

#### 3.2.3. Chiral Gel-Assisted Synthesis Method

Chiral gels can also function as chiral hosts, which create a chiral microenvironment for the system. Achiral nanomaterials can emit circularly polarized luminescence when encapsulated within chiral helical nanotubes via a co-gelation process [26,50,51,52]. For instance, the co-assembly of carbon dots and chiral gelators can successfully construct composite materials with tunable chiral luminescence, where both the luminescence intensity and direction depend on the excitation wavelength [50]. Liu et al. used chiral gelator lipid N,N′-bis(octadecyl)-l-glutamic diamide (LGAm) or its enantiomer DGAm to co-assemble with achiral perovskite nanocrystals, inducing chiral transfer and causing the nanocrystals to exhibit chiral luminescence. The direction of luminescence depends on the chirality of the lipids [51]. These studies indicate that the handedness direction of the CPL in co-gel systems can be modulated by the excitation wavelength and molecular chirality. For rare earth materials, Zhou et al. designed an organic gel (BzLG), where BzLG is an abbreviation of an organogelator containing benzimidazole moiety, N,N2032-bisoctadecyl-2-(1Hbenzimidazole-2-carbonyl)-_L_-glutamic amide [75]. They developed various chiral nanostructures by introducing metal ions, which can also coordinate with rare earth metal ions. Glutamine served as the gelation agent, while the benzimidazole group acted as the coordination unit for rare earth ions. Upon the introduction of Eu(NO_3_)_3_ or Tb(NO_3_)_3_, the material preferentially assembled into a flower-like morphology consisting of aggregated nanotubes. Conversely, when EuCl_3_ or TbCl_3_ were added, nanofibers were produced, highlighting the influence of different anions on the interaction between metal ions and the gel matrix. This interaction, in turn, enables control over the gel’s structural morphology. Additionally, achiral lanthanide-doped UCNPs (NaYF_4_:Yb/Er or NaYF_4_:Yb/Tm) can be encapsulated into chiral helical nanotubes through the procedure of co-gelation (as shown in Figure 4c) [26]. These nanomaterials display upconverted CPL with wavelength ranging from 300 nm to 850 nm.

#### 3.2.4. Chiral Silica-Assisted Synthesis Method

Chiral silicas (nanospheres, twisted nanorods, and helical ribbons, etc.) have been found to exhibit chirality at either the molecular or nanoscale level [76,77,78]. These materials are considered promising chiral templates for the development of CPL-active compounds due to their stable architecture and ease of surface functionalization [79,80,81]. Lu. et al. constructed a novel type of pure inorganic chiral nanocomposites HS@CeF_3_:RE by in situ assembly strategy [33]. Left-/right-handed helical silicas (L/R-HS) are selected as chiral hosts and RE-doped CeF_3_ nanoparticles (NPs) are selected as luminophores. As shown in Figure 4d, the left- or right-handed helical ribbon structure was used as a template for inorganic transcription to obtain HS. R-HS was successively surface-functionalized with 3-aminopropyltriethoxysilane and succinic anhydride. Rare earth ions were attracted to the HS surface through coordination and electrostatic interactions. Subsequently, NaHF_2_ was added as a fluorine source. With the aid of ultrasonic treatment, CeF_3_:TbNP was rapidly in situ assembled within 2 min, thereby preparing L/R-HS@CeF_3_:Tb nanocomposites. In addition, Duan et al. also reported an impressive upconversion CPL (UC-CPL) example from a co-assembled system composed of helical platform and UCNPs [82]. This work has suggested that an achiral molecule C_3_-symmetric benzene-1,3,5-tricarboxamid with three identical benzoic acid arms (BTABA) can form chiral nano helices through symmetry breaking. This method provides a universal approach for designing tunable UC-CPL materials, greatly expanding the research scope of CPL materials. Importantly, the co-assemblies exhibit enhanced CPL, which has been confirmed in many co-assembly systems [83].

Chiral SiO_2_ (non-helical) can also be formed through the induction of chiral organic templates. Jin et al. [79] successfully constructed a novel solid-state inorganic CPL material system containing luminescent sub-10-nanometer rare earth oxide nanoparticles (guests) and chiral silica nanofibers (hosts) by a simple two-step adsorption–calcination method. Jin et al. [84] first mixed polyethyleneimine (PEI) with chiral tartaric acid (tart) to form chiral crystal complexes (CCCs). Subsequently, they dispersed the CCC in a mixed solution of water and tetramethyl orthosilicate (TMOS), and deposited silica through hydrolysis and condensation reactions. Finally, they removed the organic template via high-temperature calcination, resulting in high-temperature-resistant chiral silica (PEI/tart@SiO_2_ nanofibers) as shown in Figure 4e. Jin’s team [79] incorporated rare earth ions into PEI/tart@SiO_2_ nanofibers. The research found that this inorganic system exhibits CD optical activity in the ultraviolet wavelength region. This work opens up new avenues for the development of CPL systems based on inorganic materials and indicates that chiral silica hosts can effectively transfer chiral information to rare earth oxides guests.

#### 3.2.5. Chiral Layered Structure-Assisted Synthesis Method

Under the influence of chiral molecules, BiOCl tends to form chiral layered nanostructures [85]. Inorganic nanostructures with a chiral layered morphology can also function as inorganic chiral hosts. Qiu et al. [34] synthesized BiOCl: 5%Yb^3+^/4%Er^3+^ (BYE) UC-CPL materials with D-sorbitol (D-Sor) (Figure 4f) using a hydrothermal method followed by calcination in air. Compared to the BiOCl material without chiral ligands (A-BYE), D-BYE exhibits a significantly stronger upconversion luminescence intensity. D-BYE demonstrates pronounced CPL properties under 980 nm laser excitation, whereas A-BYE does not. This indicates that the chiral layered structure is not only essential for the emission intensity of CPL but also enhances the photoluminescence quantum yield (PLQY). The utilization of rare earth-doped chiral layered nanomaterials offers a novel strategy for achieving UC-CPL.

### 3.3. Self-Assembly Method

**Figure 5:**
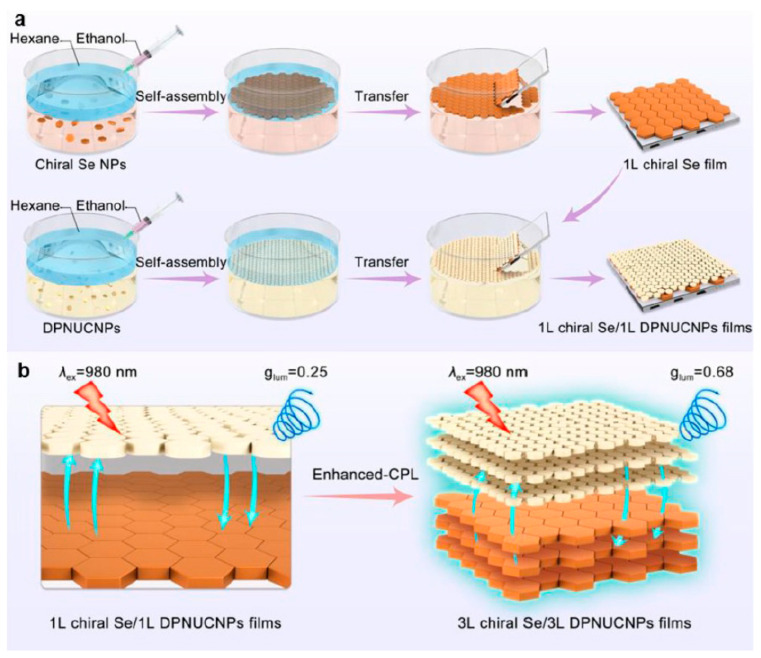
Schemes of (**a**) the preparation procedure for the 2D heterostructure films through interfacial self-assembly of D-/L-Se NPs and DPNUCNPs; (**b**) enhanced-CPL by controlling the layer number of D-/L-Se/DPNUCNPs films under 980 nm excitation [89].

The development of functional nanostructures through lanthanide-directed self-assembly has become an increasingly important area of research, given their potential applications in molecular recognition, sensing, imaging, and optical devices [56,86,87,88]. Two-dimensional heterostructure films (D-/L-Se/DPNUCNPs films) were fabricated by self-assembly at the liquid–liquid interface using D-/L-Se NPs and NaYF_4_:Yb/Tm upconversion nanoparticles (DPNUCNPs) [89]. As shown in Figure 5a, first, a monolayer D-/L-Se film (1L D-/L-Se film) was formed on a quartz substrate using the liquid–liquid interfacial assembly technique. Following this, a layer of DPNUCNPs was applied to the surface of the chiral Se film by the same method, resulting in the formation of a two-dimensional heterogeneous structure composed of 1L D-/L-Se and 1L DPNUCNPs films. Furthermore, the CPL performance of the films was optimized by controlling the assembly sequence and the number of layers as shown in Figure 5b. The basic mechanism of chirality transfer within these 2D self-assembled films is that chirality is transferred from the excited state of the chiral Se NPs to the excited state of the DPNUCNPs during the energy transfer process from DPNUCNPs to Se NPs. In addition, chiral amphiphilic Nd (III) complexes form monolayer films via the Langmuir–Blodgett technique [56].

## 4. Chiroptical Properties

Chiroptical (chiral–optical) properties refer to the optical characteristics of molecules or objects [90]. These properties arise due to the strong interaction between chiral nanomaterials and electromagnetic waves, leading to phenomena such as CD and CPL [35,91,92]. Generally, natural light from sunlight or other common sources is considered unpolarized, with its electromagnetic waves emitted and propagated randomly at various polarization angles. The polarized light can be simply classified into linearly, circularly, elliptically, and partially polarized light [93,94,95]. Experimentally, circularly polarized light can be obtained from non-polarized light by using a linear polarizer and a quarter-wave plate. However, using polarizers causes the loss of about 50% of light due to the conversion of the unpolarized light into linearly polarized light. Chiral materials exhibit unique optical activities, including CD and CPL. In the following, we will first introduce the basic concepts related to CD and CPL and then delve into the recent advancements of chiroptical properties in chiral rare earth nanomaterials.

### 4.1. Basic Concepts Related to CD and CPL

**Figure 6:**
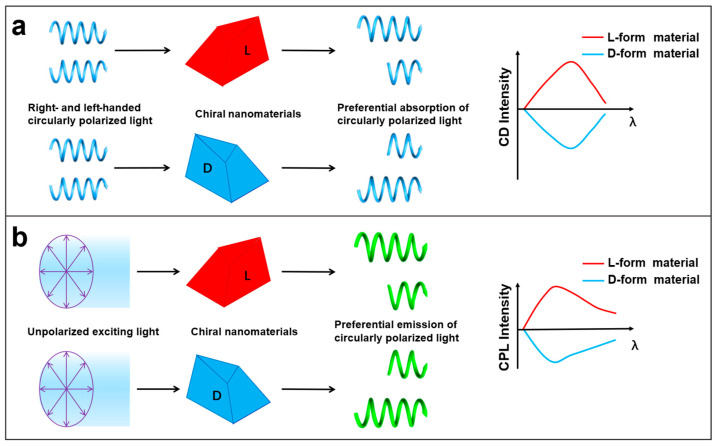
Schematic illustration of circular dichroism (CD) (**a**) and circularly polarized luminescence (CPL) (**b**).

CD and CPL involve the differential absorption and emission rates of left- and right-circularly polarized light, respectively. CD and CPL spectra are essential techniques for characterizing the optical activity of chiral materials. Figure 6 illustrates the mechanisms of CD and CPL measurements. When right- and left-handed circularly polarized light alternately pass through a chiral material (for example, an L-form material), right-handed circularly polarized light is absorbed less than left-handed circularly polarized light, as shown in Figure 6a. Conversely, when right- and left-handed circularly polarized light alternately pass through its enantiomer (D-form material), right-handed circularly polarized light is absorbed preferentially. The CD intensity is proportional to *A*_L_–*A*_R_, where *A*_L_ and *A*_R_ represent the absorbance of left- and right-handed circularly polarized light, respectively. Consequently, the CD spectrum exhibits a mirror image with positive and negative signals. On the other hand, unpolarized light is used to excite a chiral material. As shown in Figure 6b, L- and D-form materials preferentially emit right- and left-handed CPL, respectively. The CPL intensity is proportional to *I*_L_–*I*_R_, where *I*_L_ and *I*_R_ represent the intensities of left- and right-handed CPL, respectively. Positive and negative signals correspond to L- and D-form materials, respectively. CD and CPL spectra can precisely analyze the ground and excited state optical properties of materials. The complementary combination of these two techniques not only enables the quantitative assessment of the anisotropy factor (g factor) of materials but also reveals the transfer and amplification mechanism of chirality, laying a theoretical foundation for the design of high-performance optoelectronic devices [82,96].

Both the CD and CPL spectra are ultimately based on the difference in intensity of circularly polarized light. Therefore, the magnitude method of CD and CPL are similar [83]. Circular dichroism refers to the differential absorption rates of L-CPL and R-CPL by a sample. The definition of the asymmetry factor g in circular dichroism spectroscopy is $g_{\mathrm{CD}}=2(A_{L}-A_{R})/(A_{L}+A_{R})$, where *A*_L_ and *A*_R_ denote absorption coefficients of L-CPL and R-CPL, respectively. Experimentally, CD data are typically measured in terms of ellipticity (θ) (unit: deg or mdeg) [97]. This relationship of ellipticity (unit: mdeg) and *g*_CD_ is expressed in Equation (1):

(1) $$g_{\mathrm{CD}}=\frac{\mathrm{ellipticity}\times \mathrm{absorbance}}{32980}\;$$

where the “ellipticity” and the “absorbance” can be directly obtained from CD spectra. When a chiral luminescent system is excited, the emission intensities of the left and right CPL that it produces are not equal [30,98,99]. Due to the significant challenges associated with directly measuring absolute emission intensities, the luminescent dissymmetry factor ($g_{\mathrm{lum}}$) is employed to evaluate the extent of CPL. The mathematical expression for the $g_{\mathrm{lum}}$ factor is provided in Equation (2), as shown below:

(2) $$g_{\mathrm{lum}}=\frac{2\left(I_{L}-I_{R}\right)}{\left(I_{L}+I_{R}\right)}$$

where *I*_R_ and *I*_L_ represent the emission intensities of R-CPL and L-CPL, respectively. Experimentally, *g*_lum_ = [ellipticity/(32,980/ln 10)]/total fluorescence intensity at the CPL extremum [26]. The value of $g_{\mathrm{lum}}$ ranges from +2 to −2, with ±2 indicating that the system emits ideal circularly polarized light, while a $g_{\mathrm{lum}}$ value of 0 corresponds to unpolarized light [100]. Notably, when using $g_{\mathrm{CD}}$ values and $g_{\mathrm{lum}}$ values as evaluation metrics, these parameters effectively eliminate the influence of differences in sample concentration, variations in luminescence intensity, and the use of different instruments during experimentation, resulting in more accurate and reliable outcomes [29].

### 4.2. Chiroptical of Chiral Nanomaterials

**Figure 7:**
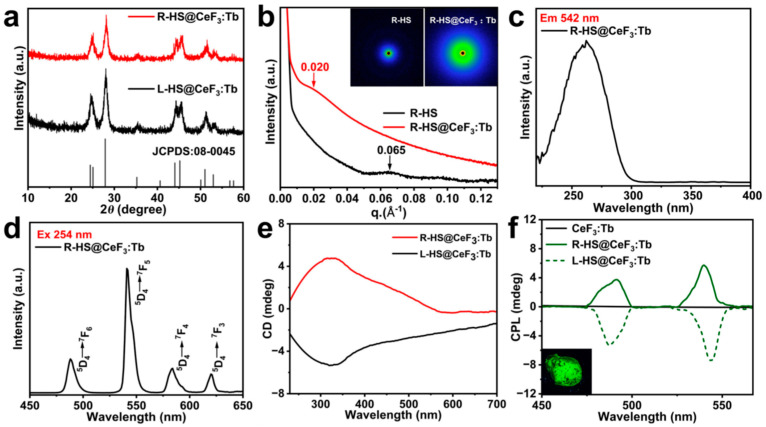
(**a**) XRD patterns of L/R-HS@CeF_3_: Tb. (**b**) SAXS patterns of R-HS and R-HS@CeF_3_: Tb nanocomposites (the insets represent two-dimensional SAXS images). PL excitation spectrum under 542 nm emission (**c**) and PL emission spectrum under 254 nm excitation (**d**) of R-HS@CeF_3_: Tb. (**e**) CD spectra of L/R-HS@CeF_3_: Tb. (**f**) CPL spectra of CeF_3_: Tb and L/R-HS@CeF_3_: Tb under 254 nm excitation (the inset represents the corresponding image of R-HS@CeF_3_: Tb upon 254 nm UV irradiation) [33].

Lu et al. developed a novel rare earth fluoride nanocomposite material and investigated its optical properties and potential applications [33]. Figure 7a,b show the XRD patterns of L/R-HS@CeF_3_: Tb and SAXS patterns of R-HS and R-HS@CeF_3_: Tb nanocomposites. As shown in Figure 7c, the broad excitation band (220 nm~300 nm) is attributed to the allowed 4f→5d transition of Ce^3+^ ions. Under excitation at 254 nm, R-HS@CeF_3_: Tb exhibited strong emission peaks of Tb^3+^ at 489, 542, 584, and 620 nm, corresponding to the ^5^D_4_→^7^F_J_ (J = 6, 5, 4, 3) transitions, respectively (Figure 7d). The CD spectra of L/R-HS@CeF_3_: Tb exhibited mirror-image symmetry, indicating that the nanoparticles were oriented in opposite directions on the surfaces of L/RHS (Figure 7e). Compared to other chiral nanomaterials, such as chiral quantum dots, it is difficult to observe the CD signals of chiral rare earth nanomaterials. This challenge may be attributed to the low concentration of rare earth nanomaterials in the complexes and the strong scattering effects [26].

Research on the CPL of chiral rare earth nanomaterials has made significant progress [101,102]. As shown in Table 1, the *g*_lum_ values range from 4.7 × 10^−3^ to 1.1. The g factor of intrinsic chiral rare earth nanomaterials is generally larger than that of non-intrinsic nanomaterials, which may be related to the degree of chiral assembly. The magnitude of *g*_lum_ can be controlled by adjusting the glycerol content [74], the number of film layers [89], and the incident light [66]. For example, the CPL activity of self-assembled films of chiral selenium nanoparticles can be effectively enhanced by controlling the assembly sequence and the number of layers, reaching a maximum value of 0.68 [89]. Additionally, by introducing stimulus-responsive groups or materials, the CPL properties can be made responsive to external conditions such as temperature and humidity [74]. Furthermore, composite materials composed of perovskite and chiral lanthanide MOFs can respond to various stimuli, including chemical substances, temperature, and light, enabling reversible switching of CPL [58].

The color of CPL in chiral rare earth nanomaterials can be tuned by varying the doped ions or by applying an external stimulus, such as glycerol [32,33,74]. For example, phosphate nanowires of LaPO_4_, GdPO_4_, and TbPO_4_ doped with Eu^3+^, Tb^3+^, or Tm^3+^, as well as rare earth-doped CeF_3_ nanoparticles assembled within helical silica, exhibit CPL emissions in different colors [32,33]. This tunability is primarily achieved by adjusting the type and concentration of rare earth ions, which controls the CPL emission wavelength. Additionally, chiral europium halide (R/S-3BrMBA)_3_EuCl_6_ demonstrates magnetic field-tunable red CPL at room temperature, with its degree of polarization modulated by an external magnetic field [105]. Furthermore, the CPL emission intensity of chiral nanomaterials is generally higher than that of their non-chiral counterparts [34].

Some chiral rare earth nanomaterials exhibit excellent thermal stability, maintaining CPL activity even under high-temperature conditions [32,33]. These studies indicate that chiral inorganic nanomaterials hold significant potential for applications in the CPL field. By integrating chiral assembly strategies, rare earth-doped luminescent materials, and photonic crystal structures, it is possible to fabricate efficient, tunable, and stimulus-responsive CPL materials.

## 5. Potential Applications

Chiral substances play a crucial role in biological systems. Chiral inorganic nanomaterials have similar size, charge, surface properties, and morphology to natural chiral nanomaterials, but usually exhibit extraordinary properties, such as high g factor values and enantiomeric configuration [28]. Chiral nanomaterials with intrinsic chirality or spatial asymmetry at the nanoscale are currently in the limelight of both fundamental research and diverse important potential applications, such as biosensing, drug delivery, early diagnosis, bioimaging, and disease treatment. In the following, we will elaborate the application prospects of chiral rare earth nanomaterials in circularly polarized luminescence devices, information encryption, biosensing, bioimaging, and disease diagnosis and treatment.

### 5.1. Circularly Polarized Light-Emitting Devices

CPL materials have broad application prospects in fields such as information security, 3D displays, and optoelectronic devices. Currently, most reported CPL materials are organic molecular systems, which are sensitive to environmental factors and exhibit poor stability. Inorganic nanomaterials, on the other hand, offer better control over optical performance and higher chemical stability, making them a hotspot in CPL material research. By doping different kinds of rare earth ions (such as Eu^3+^, Tb^3+^, Tm^3+^) into the crystal structure, the emission colors of CPL can be tuned. Wang et al. successfully prepared chiral LaPO_4_:RE^3+^ (RE = Eu, Tb, Tm) nanowires, achieving CPL emissions in red, green, and blue colors [32]. The presence of atomic-scale chirality ensures that CPL emissions are unaffected by molecular ligands and exhibit excellent thermal stability, maintaining stable luminescence even at high temperatures of 300 °C. Furthermore, they dispersed the nanowires into a polymer matrix to create transparent, flexible CPL films, laying the foundation for practical applications of CPL materials.

**Figure 8:**
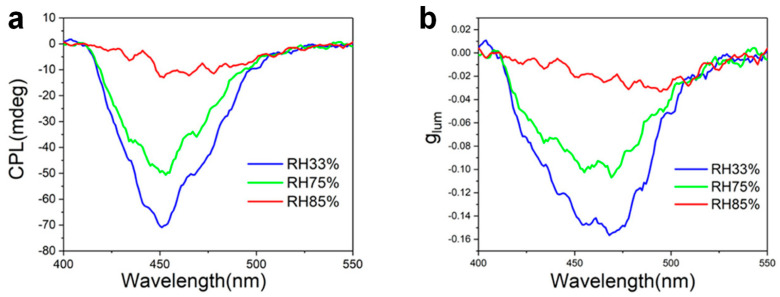
Humidity-dependent UC-CPL observed in the glycerol-integrated photonic film. CPL spectra (**a**) and the corresponding g_lum_ distribution curves (**b**) of film under varying relative humidity (RH) conditions [74].

Li et al. constructed chiral photonic films using cellulose nanocrystals (CNCs) and discovered that glycerol-based composite films exhibit humidity-responsive UC-CPL at blue light wavelengths, with glum values varying with relative humidity as shown in Figure 8a,b [74]. This humidity-responsive UC-CPL material offers new insights for the development of intelligent CPL devices. This opens new avenues for designing high-stability, tunable CPL materials, facilitates a deeper understanding of inorganic chiral information, and provides fresh insights for the development of novel optoelectronic devices with outstanding CPL properties.

### 5.2. Optical Anti-Counterfeiting and Information Encryption

CPL can be a type of luminescence generated by the intrinsic chirality of luminescent molecules or complexes. It encodes the “fingerprints” of chiral molecules, which cannot be replicated. Conventional CPL spectrometers are slow and costly, limiting their application in security inks. In recent years, the emergence of next-generation fast CPL spectrometers and CPL microscopes has made rapid verification and imaging of CPL security inks possible. Lanthanide metal complexes hold great potential in security inks, particularly in the realm of CPL applications. With the continuous advancement of CPL analysis and reading technologies, CPL-active lanthanide metal security inks are expected to become an important component of the next generation of security inks [106]. Additionally, Chiral lanthanide lumino-glass (such as europium complexes) exhibit switchable CPL patterns under UV light, leading to new applications of CPL materials as security inks [107].

**Figure 9:**
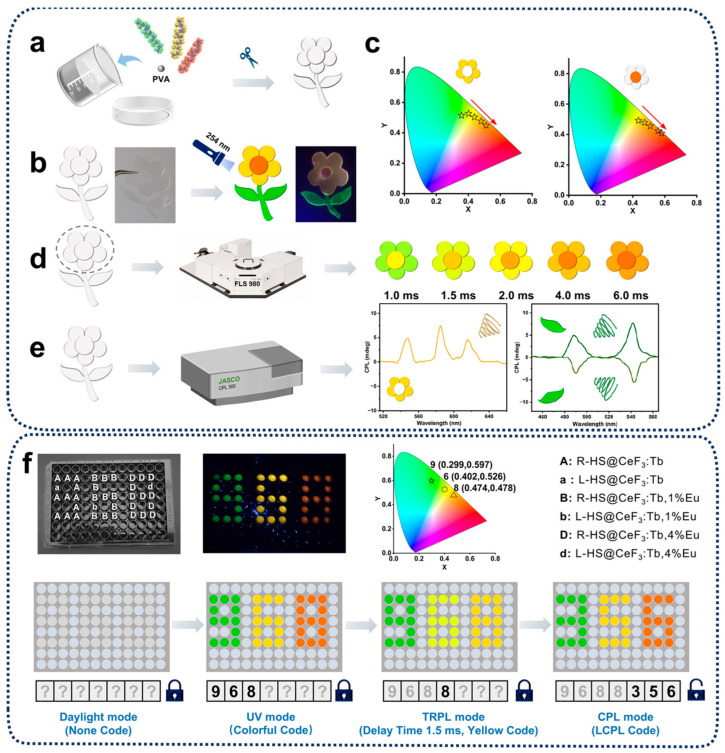
The illustration shows (**a**–**e**) anti-counterfeiting patterns and (**f**) multilayer optical information encryption codes based on L/RHS@CeF_3_:RE nanocomposites. (**a**) Preparation process of luminescent plant pattern. (**b**) Photographs of patterns under daylight and UV light. (**c**) Time-dependent CIE chromaticity diagram for petal (left) and stamen (right). (**d**) Dynamic PL changes in flower stamen and petal. (**e**) CPL spectra of pattern under 254 nm UV excitation. (**f**) Demonstration of the step-by-step decryption of multicolor codes through the modes of UV light, TRPL, and CPL. Insets show the arrayed nanocomposites in a 96-well plate, the corresponding photograph under 254 nm irradiation, and the CIE chromaticity diagram at a delay time of 1.5 ms [33].

Lu et al. reported a novel inorganic chiral nanocomposite made of helical silica HS and rare earth fluoride nanoparticles, exhibiting multicolor CPL and time-resolved photoluminescence (TRPL) properties for advanced anti-counterfeiting applications as shown in Figure 9 [33]. They mixed different nanocomposites with PVA to create films and fabricated plant patterns. Under daylight, the patterns are transparent; however, when exposed to UV light, the patterns emit different colors of light and exhibit dynamic and chiral signals, enabling advanced anti-counterfeiting features. Additionally, they delved into the application potential of these nanocomposites for information encryption. By incorporating different nanocomposites into a 96-well plate, they created multicolor codes that can be decrypted step-by-step through various decryption methods (UV light, TRPL, CPL), achieving multilayer optical encryption. Additionally, Hao et al. reported that the chiral Se/DPNUCNPs films were also patterned for encryption applications [89]. Notably, this upconversion CPL of chiral Se/DPNUCNPs films were excited by near-infrared irradiation (980 nm), which holds significant potential for future applications in encryption inks due to its hard replication.

### 5.3. Drug Delivery, Therapy, and Bioimaging

Chiral rare earth nanomaterials have good biocompatibility and can be used in biomedical applications such as bioimaging and photodynamic therapy. In recent years, a large number of studies have demonstrated that rare earth-doped nanomaterials have shown significant application value in the biomedical field, especially in bioimaging and photodynamic therapy [108,109,110,111]. These studies have confirmed their advantages from different perspectives, for example, the unique advantages of upconversion nanoparticles in bioimaging and antibacterial treatment [108,109], diagnosis, and treatment [111]. Wang et al. introduced some applications of chiral inorganic nanomaterials, including the treatment of Alzheimer’s disease, cancer, and viral infections, demonstrating their significant potential in biomedicine and bioengineering [112]. Chiral rare earth complexes can selectively bind to specific biomolecules, such as proteins and nucleic acids, through their CPL properties [113]. Additionally, by leveraging the CPL characteristics of chiral rare earth materials, image contrast can be enhanced, thereby improving the resolution of biomedical imaging. For example, chiral BiOCl:Er^3+^/Yb^3+^ nanostructures show promise for biological imaging due to their enhanced upconversion luminescence [34].

**Figure 10:**
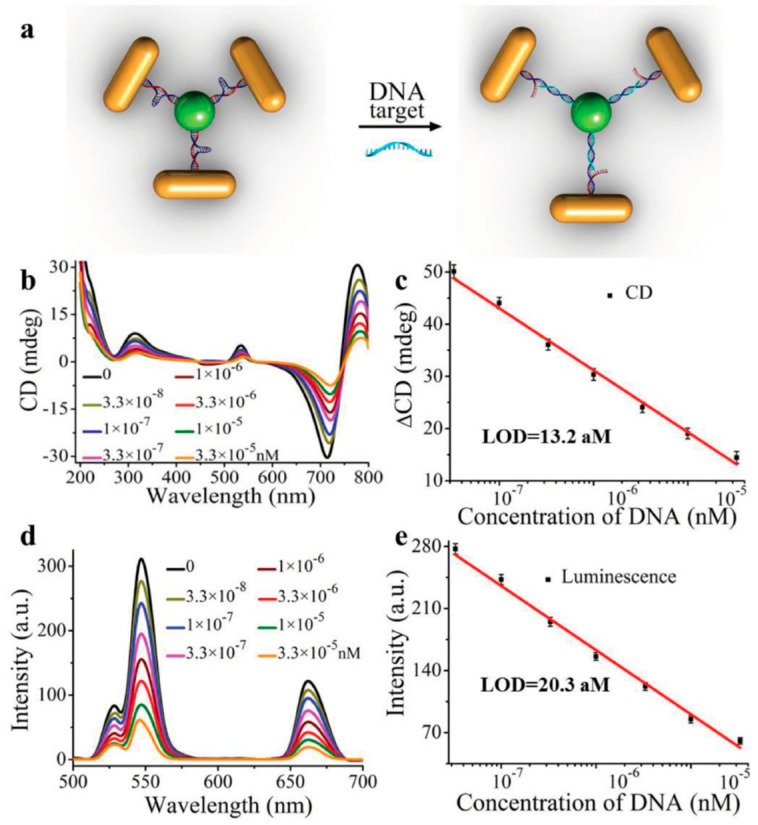
(**a**) Schematic illustration for DNA biosensing. The CD (**b**) and upconversion luminescence (**d**) curves with increasing concentrations of DNA solution. The CD (**c**) and upconversion luminescent (**e**) calibration curves for DNA detection [114].

Kuang et al. developed a gold nanorod (NR)-UCNP tetramer assembly featuring tunable optical properties and dual-mode biosensing capabilities [114]. When the oligonucleotide targets are present, the hairpin-like DNA strands within the tetramers extend as shown in Figure 10a. This extension increases the gap length, resulting in a decrease in both upconversion luminescence and CD signal intensities as shown in Figure 10b–e. Owing to the strong optical activity and enhanced luminescence of the tetramer, it can be employed for dual-mode biosensing, simultaneously detecting upconversion luminescence, and circular dichroism signals. This method can detect DNA at concentrations as low as the attomolar level and demonstrates high specificity and reliability.

## 6. Summary and Outlook

This paper reviews the research progress on chiral rare earth nanomaterials. Firstly, it introduces the chirality origins and synthesis methods for these materials in detail. Researchers have achieved controllable design of the morphology, size, and chiral features of rare earth nanomaterials by carefully adjusting the synthesis processes. For intrinsic chiral rare earth nanomaterials, hydrothermal/solvothermal methods are commonly used, utilizing rare earth ions with excellent luminescent properties (such as Eu^3+^, Tb^3+^, and Tm^3+^), rare earth oxides, and upconversion nanoparticles. These intrinsic chiral rare earth nanomaterials not only exhibit tunable luminescent colors but also demonstrate good thermal stability. In terms of non-intrinsic chiral hosts, chiral MOFs, chiral liquid-crystalline structures, chiral helical structures, chiral organic gels, and chiral SiO_2_ provide favorable chiral environments for luminescent guest species. When luminescent guests are assembled along the spatial structure of the chiral hosts, chiral composites can be constructed. Next, the optical properties of chiral rare earth nanomaterials are introduced, including CD and CPL spectra. The *g*_CD_-value and g_lum_-value eliminate the influences of concentration and luminescence intensity, making them significant for the study of the optical properties of chiral materials. Finally, based on the excellent optical performance of chiral rare earth nanomaterials, we introduce their potential applications in CPL devices, optical anti-counterfeiting and information encryption, drug delivery, and bioimaging.

Despite significant progress in the research of chiral rare earth nanomaterials, several challenges remain. Key issues include achieving large-scale, cost-effective synthesis of high-quality materials, further enhancing chiroptical properties, and addressing their stability and biosafety in complex environments. Further research is essential to improve the luminescence efficiency [115,116] and CPL intensity of chiral rare earth materials while expanding their range of applications. For instance, optimizing chiral templates and controlling the coordination environment of rare earth ions could significantly enhance CPL signal strength. Additionally, to facilitate their use in biomedical applications, it is crucial to improve water solubility and develop more water-soluble chiral groups suitable for aqueous solutions. The development and application of CPL in the biomedical field remain in their early stages. To achieve clinical translation, technical bottlenecks must be further addressed. For example, future efforts could focus on preparing chiral rare earth nanomaterials with highly responsive CPL to biological small molecules to overcome current limitations. Such advancements will contribute to building a medical platform that integrates diagnosis and real-time treatment monitoring, thereby enabling visualization of the treatment process.

## Figures and Tables

**Table 1:** Summary of the maximum representative *g*_lum_ in this review. (BTABA = benzene-1,3,5-tricarboxamid with three identical benzoic acid arms, NCs = nanocrystals, DAEC = diarylethene derivative).

Chirality	Chirality Origin	CPL-Active System	Maximum *g*_lum_	Reference
Intrinsic	Chiral lattice	GdPO_4_:Eu^3+^ phosphate	1.1 × 10^−1^	[32]
	Chiral lattice	NaYF_4_:Eu^3+^ phosphate	4 × 10^−1^	[41]
Non-intrinsic	Chiral helical nanotubes	NaYF_4_:Yb/Tm UCNPs	5.48 × 10^−3^	[26]
	Helical silica	CeF_3_:Tb^3+^ nanoparticles	4.7 × 10^−3^	[33]
	Chiral MOF	NaYF_4_:Yb/Er UCNPs	1.2 × 10^−2^	[65]
	Cellulose nanocrystal	NaYF_4_:Tm/Yb UCNPs	1.56 × 10^−1^	[74]
	Chiral nematic liquid crystal	UCNPs and CsPbBr_3_ NCs	1.1	[103]
	Chiral MOF	DAEC and UCNP-Tm	7.8 × 10^−2^	[66]
	Chiral CsPbBr_3_ NCs	NaYF_4_:Yb/Tm UCNPs	5.0 × 10^−3^	[104]
	BTABA	NaYF_4_:Yb/Er nanoparticles	1.2 × 10^−2^	[82]
